# Whole genome sequencing of 51 breast cancers reveals that tumors are devoid of bovine leukemia virus DNA

**DOI:** 10.1186/s12977-016-0308-3

**Published:** 2016-11-04

**Authors:** Nicolas A. Gillet, Luc Willems

**Affiliations:** 1 Molecular and Cellular Epigenetics, Interdisciplinary Cluster for Applied Genoproteomics (GIGA), University of Liège (ULg), B34, 1 Avenue de l’Hôpital, 4000 Sart-Tilman Liège, Belgium; 2 Molecular and Cellular Biology, Gembloux Agro-Bio TechUniversity of Liège (ULg), 13 Avenue Maréchal Juin, 5030 Gembloux, Belgium

**Keywords:** Breast cancer, Bovine leukemia virus, BLV

## Abstract

Controversy exists regarding the association of bovine leukemia virus (BLV) and breast cancer. PCR-based experimental evidence indicates that BLV DNA is present in breast tissue and that as many as 37% of cancer cases may be attributable to viral exposure. Since this association might have major consequences for human health, we evaluated 51 whole genomes of breast cancer samples for the presence of BLV DNA. Among 32 billion sequencing reads retrieved from the NCBI database of genotype and phenotype, none mapped on different strains of the BLV genome. Controls for sequence divergence and proviral loads further validated the approach. This unbiased analysis thus excludes a clonal insertion of BLV in breast tumor cells and strongly argues against an association between BLV and breast cancer.

## Background

BLV naturally infects cattle, water buffalo, yak and zebu [1–4]. Sporadic infections with BLV have occasionally been reported in other species like alpaca [5]. Experimentally, BLV can also be transmitted to a number of species including sheep [6], goats [6], rats [7] and rabbits [8]. BLV infection causes B cell lymphocytosis, leukemia and/or lymphoma in natural and some experimental hosts [1]. There is also controversial evidence suggesting that BLV might infect humans: (1) antibodies against the BLV capsid were detected in 74% of human sera from the Berkeley Community, California [9], (2) BLV DNA was detected in breast tissues using PCR [10–12]. Based on a positive correlation between the rates of BLV infection and tumor frequencies (36–59% compared to 29–45% in normal tissue), as many as 37% of breast cancer cases may be attributable to BLV exposure [12].

Although these observations initiated some skepticism within the scientific community [13], the potential consequences for human health clearly require further investigation.

## Results and discussion

**Fig. 1:**
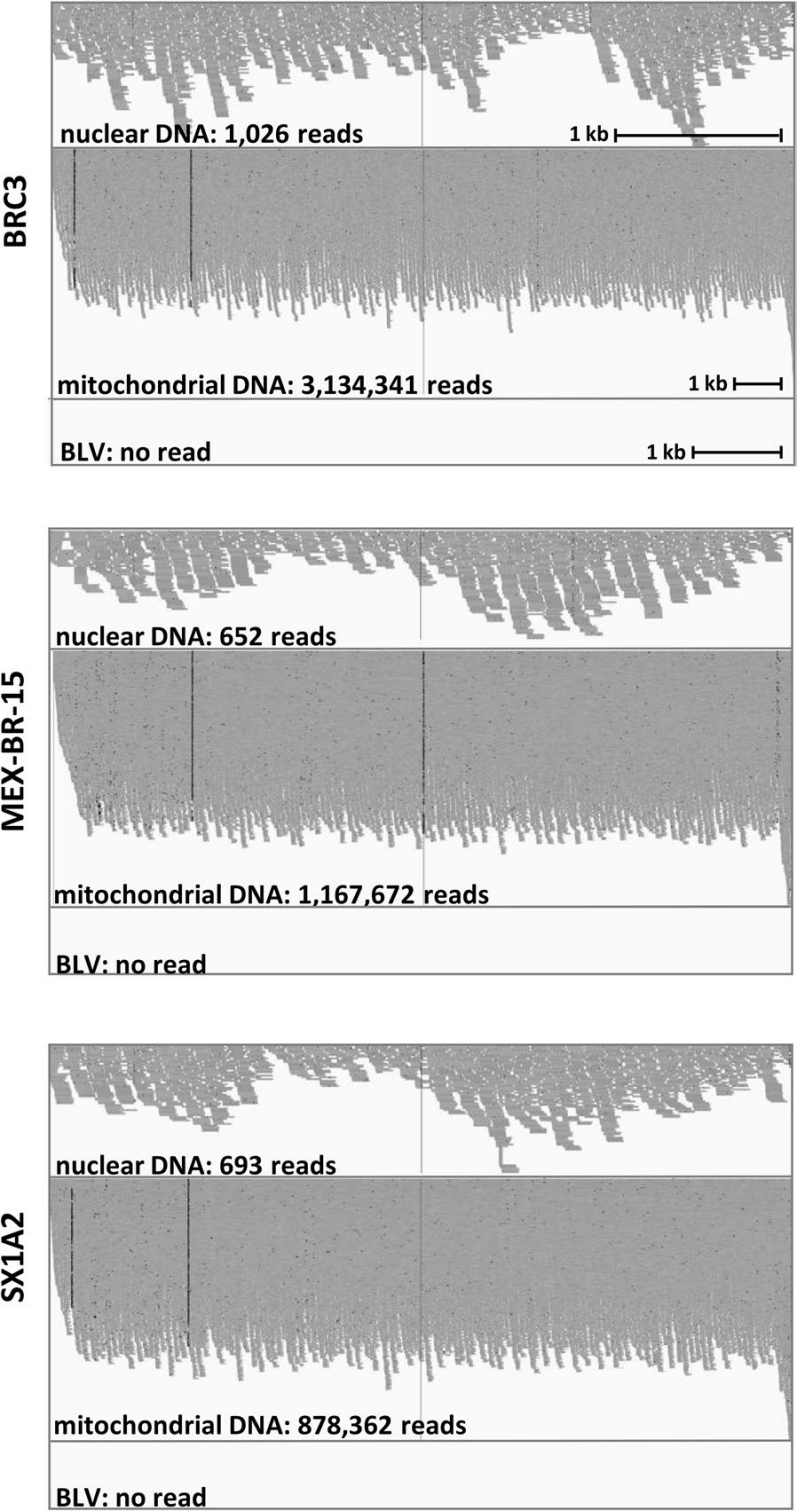
Representative alignment of dbGaP sequencing reads to human and BLV DNA. Breast cancer patients were BRC3 from USA (study phs000472), MEX-BR-15 from Mexico and SX1A2 from Vietnam (study phs000369). Aligned reads were visualized using integrative genomics viewer (IGV)

**Fig. 2:**
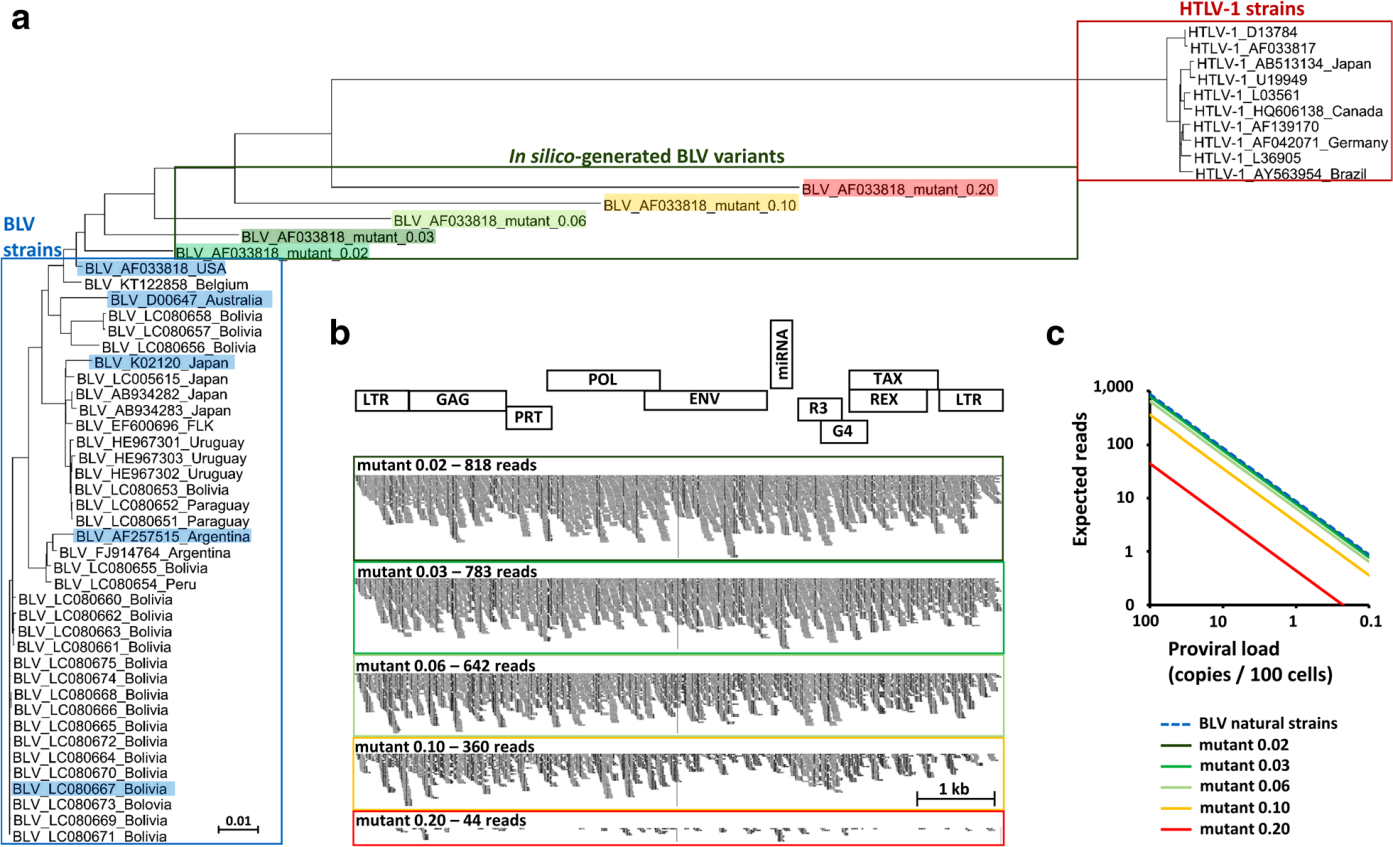
Analysis of sequence variation and proviral load in sequence alignments. **a** Neighbour-joining phylogenetic tree of BLV and HTLV-1 genomes. **b** Using the ART simulation tool (NIH), Illumina-like 100 bp paired-reads were generated *in silico* from the mutants. 880 simulated reads were probed for alignment on BLV AF033818 using Bowtie2 and visualized using IGV. **c** Correlation between proviral loads and predicted number of reads

To avoid potential experimental artifacts associated with DNA amplification techniques, we directly analyzed whole genomes of breast tumors and adjacent tissues. After retrieval of raw DNA sequences from the NCBI dbGaP [14, 15], paired-reads were probed for alignment on different BLV strains using Bowtie2. As a positive control, a nuclear DNA fragment (chr12: 53,959,600–53,964,000) devoid of repeated sequences that would lead to an overestimation of aligned reads and set to 4.4 kb to fit with the monoploid 8.8 kb BLV genome was selected from the human genome. Alignment of 51 breast tumors genomes on the nuclear control sequence identified between 283 and 1287 paired-reads (illustrated on Fig. 1 and summarized on Table 1). In contrast, no homology was found with 5 different BLV subtypes (highlighted in blue on the phylogenic tree of Fig. 2a). In 19 biopsies adjacent to the breast tumors, 386–1197 paired-reads aligned onto the nuclear DNA sequence whereas none mapped on BLV (Table 1). All DNA samples contained extranuclear DNA as indicated by alignment of a control mitochondrial sequence (NC_012920) (Table 1).

**Table 1:** Absence of BLV DNA in 51 whole genomes of breast tumors

Subject ID	Country	Age	Diagnosis	Sample type	Grade	HER2 status	ER status	PR status	Total no of reads	No. of reads that align on						
										Control DNA (nuclear)	Control DNA (mitochondrial)	BLV_AF033818	BLV_AF257515	BLV_D00647	BLV_K02021	BLV_LC080667
MEX-BR-106	Mexico	42	IDC	Tumor	II	−	+	+	583,906,975	669	396,239	0	0	0	0	0
MEX-BR-116	Mexico	92	IDC	Tumor	III	−	+	−	577,618,196	796	1,166,916	0	0	0	0	0
MEX-BR-15	Mexico	45	IDC	Tumor	II	+	−	+	571,043,227	652	1,167,672	0	0	0	0	0
MEX-BR-154	Mexico	52	IDC	Tumor	III	−	+	+	700,630,351	811	400,383	0	0	0	0	0
MEX-BR-165	Mexico	42	IDC	Tumor	II	−	+	+	757,323,566	737	742,646	0	0	0	0	0
MEX-BR-198	Mexico	44	IDC	Tumor	II	−	+	+	745,509,529	1019	1,264,555	0	0	0	0	0
MEX-BR-50	Mexico	47	IDC	Tumor	II	−	+	+	605,198,587	653	958,812	0	0	0	0	0
MEX-BR-82	Mexico	59	IDC	Tumor	II	−	+	−	681,881,066	687	547,863	0	0	0	0	0
BRC12	USA	81	IDC	Tumor	II	−	U	U	548,255,169	745	1,113,306	0	0	0	0	0
BRC13	USA	51	IDC	Tumor	III	−	7	U	587,461,482	686	1,106,780	0	0	0	0	0
BRC14	USA	86	IDC	Tumor	III	−	7	U	755,094,207	899	1,469,976	0	0	0	0	0
BRC15	USA	83	IDC	Tumor	II	−	7	U	758,784,262	934	2,327,824	0	0	0	0	0
BRC16	USA	61	IDC	Tumor	III	−	7	U	821,134,040	1287	2,084,782	0	0	0	0	0
BRC18	USA	85	IDC	Tumor	I	−	8	U	568,355,455	677	1,395,823	0	0	0	0	0
BRC19	USA	75	IDC	Tumor	II	−	8	U	596,337,842	747	1,648,870	0	0	0	0	0
BRC20	USA	61	IDC	Tumor	III	−	4	U	507,651,900	570	1,026,830	0	0	0	0	0
BRC21	USA	73	IDC	Tumor	I	−	7	U	719,742,122	817	1,710,010	0	0	0	0	0
BRC22	USA	64	ILC	Tumor	I	−	6	U	608,469,920	708	953,100	0	0	0	0	0
BRC23	USA	68	IDC	Tumor	I	−	7	U	613,481,215	687	1,272,519	0	0	0	0	0
BRC24	USA	51	IDC	Tumor	II	−	7	U	656,115,800	721	1,980,030	0	0	0	0	0
BRC25	USA	52	IDC	Tumor	II	−	5	U	583,560,227	712	580,203	0	0	0	0	0
BRC28	USA	52	IDC	Tumor	I	−	7	U	664,667,777	781	973,990	0	0	0	0	0
BRC29	USA	74	IDC	Tumor	III	−	6	U	785,019,563	596	2,085,482	0	0	0	0	0
BRC3	USA	62	IDC	Tumor	II	−	8	U	695,174,967	1026	3,134,341	0	0	0	0	0
BRC30	USA	60	ILC	Tumor	II	−	5	U	663,769,744	794	1,442,014	0	0	0	0	0
BRC31	USA	66	IDC	Tumor	II	−	6	U	734,384,352	1028	1,415,996	0	0	0	0	0
BRC32	USA	54	IDC	Tumor	I	−	7	U	643,884,178	703	1,404,436	0	0	0	0	0
BRC33	USA	83	IDC	Tumor	II	−	8	U	660,668,877	819	1,284,599	0	0	0	0	0
BRC34	USA	79	IDC	Tumor	I	−	7	U	572,861,930	704	1,499,414	0	0	0	0	0
BRC35	USA	76	IDC	Tumor	II	−	6	U	543,480,474	697	1,709,943	0	0	0	0	0
BRC36	USA	68	IDC	Tumor	II	−	7	U	706,448,348	804	1,501,763	0	0	0	0	0
BRC40	USA	66	IDC	Tumor	I	−	8	U	600,847,516	690	1,686,112	0	0	0	0	0
BRC41	USA	55	IDC	Tumor	II	−	8	U	689,312,217	812	3,735,591	0	0	0	0	0
BRC42	USA	74	IDC	Tumor	II	−	U	U	684,312,302	685	1,308,948	0	0	0	0	0
BRC44	USA	64	IDC	Tumor	II	−	7	U	717,390,251	891	1,430,064	0	0	0	0	0
BRC47	USA	54	IDC	Tumor	III	−	5	U	580,674,755	865	960,944	0	0	0	0	0
BRC48	USA	66	IDC	Tumor	II	−	6	U	782,262,353	783	1,236,102	0	0	0	0	0
BRC49	USA	56	IDC	Tumor	II	−	8	U	577,656,003	559	881,804	0	0	0	0	0
BRC5	USA	72	IDC	Tumor	II	−	7	U	762,026,860	1155	2,462,819	0	0	0	0	0
BRC50	USA	78	ILC	Tumor	I	−	4	U	661,525,693	792	357,915	0	0	0	0	0
BRC7	USA	78	IDC	Tumor	II	−	8	U	455,727,994	795	580,484	0	0	0	0	0
BRC8	USA	87	IDC	Tumor	I	−	8	U	518,548,285	628	1,394,439	0	0	0	0	0
BRC9	USA	65	ILC	Tumor	II	−	8	U	516,702,802	697	1,759,444	0	0	0	0	0
9DDA1	Vietnam	60	IDC	Tumor	III	U	U	U	706,450,950	759	1,109,340	0	0	0	0	0
9P4X9	Vietnam	54	IDC	Tumor	III	U	U	U	610,913,537	778	619,066	0	0	0	0	0
9YBUF	Vietnam	52	IDC	Tumor	III	U	U	U	595,959,881	616	788,058	0	0	0	0	0
CI5PD	Vietnam	51	IDC	Tumor	III	U	U	U	572,612,309	626	786,787	0	0	0	0	0
FYGW6	Vietnam	38	IDC	Tumor	III	U	U	U	238,201,059	282	221,942	0	0	0	0	0
GT33 V	Vietnam	52	IDC	Tumor	III	U	U	U	548,640,325	604	766,320	0	0	0	0	0
SX1A2	Vietnam	53	IDC	Tumor	III	U	+	+	598,405,143	693	1,002,577	0	0	0	0	0
UQWDS	Vietnam	35	IDC	Tumor	III	U	−	−	596,126,825	665	1,285,884	0	0	0	0	0
9DDA1	Vietnam	60	IDC	Normal	III	U	U	U	691,060,649	797	1,122,133	0	0	0	0	0
9P4X9	Vietnam	54	IDC	Normal	III	U	U	U	601,815,791	664	694,153	0	0	0	0	0
9YBUF	Vietnam	52	IDC	Normal	III	U	U	U	593,968,922	646	1,202,175	0	0	0	0	0
CI5PD	Vietnam	51	IDC	Normal	III	U	U	U	566,065,567	595	911,133	0	0	0	0	0
FYGW6	Vietnam	38	IDC	Normal	III	U	U	U	337,274,647	386	361,063	0	0	0	0	0
GT33 V	Vietnam	52	IDC	Normal	III	U	U	U	581,403,783	652	1,189,003	0	0	0	0	0
SX1A2	Vietnam	53	IDC	Normal	III	U	+	+	608,739,604	700	878,362	0	0	0	0	0
UQWDS	Vietnam	35	IDC	Normal	III	U	−	−	590,387,671	685	829,847	0	0	0	0	0
MEX-BR-106	Mexico	42	IDC	Normal	II	−	+	+	539,137,287	526	351,034	0	0	0	0	0
MEX-BR-116	Mexico	92	IDC	Normal	III	−	+	−	513,833,151	520	258,287	0	0	0	0	0
MEX-BR-123	Mexico	71	IDC	Normal	III	−	+	U	668,026,494	761	515,501	0	0	0	0	0
MEX-BR-15	Mexico	45	IDC	Normal	II	+	−	+	592,958,041	670	756,778	0	0	0	0	0
MEX-BR-154	Mexico	52	IDC	Normal	III	−	+	+	670,289,201	929	817,446	0	0	0	0	0
MEX-BR-165	Mexico	42	IDC	Normal	II	−	+	+	712,308,425	706	537,516	0	0	0	0	0
MEX-BR-198	Mexico	44	IDC	Normal	II	−	+	+	726,225,752	831	216,109	0	0	0	0	0
MEX-BR-200	Mexico	42	IDC	Normal	II	−	+	+	767,097,542	1197	279,031	0	0	0	0	0
MEX-BR-28	Mexico	79	MC	Normal	II	−	+	+	588,561,634	607	215,022	0	0	0	0	0
MEX-BR-50	Mexico	47	IDC	Normal	II	−	+	+	551,537,695	618	394,842	0	0	0	0	0
MEX-BR-82	Mexico	59	IDC	Normal	II	−	+	−	608,849,308	719	385,789	0	0	0	0	0

Whole genome sequencing data from 51 breast tumors and 19 normal adjacent breast tissues were downloaded from the NCBI dbGaP. Hundreds of millions of paired-reads per sample were probed for alignment on different BLV strains and on nuclear and mitochondrial human control sequences

*IDC* infiltrating ductal carcinoma, *ILC* infiltrating lobular carcinoma, *MC* mixed carcinoma, *U* unknown

Although no paired-read corresponding to five different BLV variants could be identified, the possibility remains that extensive sequence variability impaired detection. On average, the whole genome sequencing procedure generated 660 million reads per sample. Given that the BLV provirus length is 8.8 kb and that a normal human diploid genome is 6.6 billion base pairs, the average number of reads that would be generated by a 8.8 kb-long monoploid sequence is 880 (660,000,000/6600,000,000 × 8800). Providing that the BLV provirus is integrated in a single copy per cell, the whole genome sequencing procedure would thus generate 880 reads on average. If the strain in the sample diverges from the five reference sequences, a fraction of the reads would not be retrieved. Therefore, BLV variants were artificially generated *in silico* by introducing 2, 3, 6, 10 and 20% nucleotide changes in reference AF033818 (mutants 0.02, 0.03, 0.06, 0.10 and 0.20, respectively). Phylogenetic analysis of Fig. 2a illustrates that *in silico* generated divergence far exceeds the maximal natural sequence variations observed worldwide [16]. 880 Illumina-like reads were then simulated from these *in silico* variants using ART simulation tool and mapped on BLV genome AF033818. Most reads (818 of 880) generated from mutant 0.02 aligned on reference sequence AF033818 (Fig. 2b). Even the highly divergent mutant 0.10 still aligned 41% of its 880 reads on the reference. Up to 20% divergence in mutant 0.20 was required to significantly impair detection, although BLV specific reads were still identified (Fig. 2b).

Whole genome analysis thus excludes clonal integration of natural and highly divergent BLV strains in breast tumors. Since only a small proportion of cells may carry the provirus, the sensitivity of the analysis was correlated to the proviral loads. Any natural BLV variant that would infect 10% of the tumor cells is expected to generate about 100 reads (Fig. 2c, dotted blue line). The number of expected reads decreases along with the percentage of infected cells to reach approximately one read with a proviral load of 0.1% (Fig. 2c, dotted blue line). Considering a 59% prevalence of breast tumors positive for BLV [12], 30 samples out of our 51 should be positive. Even with an individual proviral load around 0.1%, this should make about 30 reads (on average one per patient) mapping on BLV, whereas none were found.

Using whole genome analysis, we concluded that there is no evidence for a single BLV-specific or even related sequence. The discrepancies and limitations of this report and others pertain to:

1. *The origin of the samples* It is indeed possible that tumor biopsies from previous studies originating from US [11, 12] and Colombia [10] significantly differ from those reported in the dbGaP NCBI database. Even if we restrict our observations on US originating samples (n = 35), the discrepancy remains highly significant. Indeed, Buehring reported 67 breast tumors positive for BLV over 114 cases [12] whereas we found none over 35 cases (the p value for fisher test is 1.12 × 10^−6^).
2. *The DNA extraction technique* In situ PCR suggested that BLV proviral DNA is localized in the cytoplasm [11, 12]. Analysis of mitochondria-specific sequences (Table 1) shows that dbGaP NCBI database includes reads corresponding to 16 kb-long, circular and extranuclear mitochondrial DNA.
3. *The strain divergence* Artificial *in silico* simulation of highly divergent mutants still identified BLV specific reads (Fig. 2b). Since nucleotide substitutions among BLV strains worldwide are limited to 2.3% [16], it remains questionable whether these mutants still belong to the same species. Further analysis show that breast tumor genomes do not map on HTLV-1 sequences (data not shown). Why BLV-conserved sequences were previously identified by PCR remains an enigma.
4. *Viral expression* Although BLV is expressed at trace levels in the bovine species, the p24 viral capsid protein was detected in 5% of breast tumors [12]. This observation is inconsistent with RNASeq analysis of 154.7 billion of transcriptome sequencing reads from The Cancer Genome Atlas Research Network [17, 18].

Our present study based on whole genome analysis excludes a clonal insertion of BLV in tumor cells and does not support converging lines of evidence which previously suggested an association between BLV infection and breast cancer.

## Methods

Raw DNA sequences from whole genomes of breast tumors and normal breast tissues adjacent the tumor were retrieved from the NCBI database of genotype and phenotype (dbGaP). These sequences were extracted from two studies: (1) estrogen receptor positive breast cancer: aromatase inhibitor response study (accession number phs000472) [14] and (2) sequence analysis of mutations and translocations across breast cancer subtypes (accession number phs000369) [15]. Archive files were downloaded with prefetch v2.5.7 and sequencing reads were extracted with fastdump v2.5.7 using “split-3” option to separate paired reads and single reads (NCBI SRA Toolkit). Paired reads were probed for alignment on different BLV variants (accession numbers: AF033818, AF275515, D00647, K02120, LC080667) and, as positive control, on human genomic sequences using Bowtie2 (version 2.2.5). We used the “very-sensitive” option of Bowtie2 to maximize the likelihood of viral detection. Analyses were performed on computing cluster running on Linux OS. BLV divergent sequences were created *in silico* by introducing substitutions, deletions or insertions with equal probabilities in 2, 3, 6, 10 and 20% of the reference AF033818 (mutants 0.02, 0.03, 0.06, 0.10 and 0.20, respectively). Neighbor-joining phylogenetic tree was elaborated using Clustal Omega (EMBL-EBI) and visualized by Dendroscope 3. Illumina-like paired-reads were generated from the BLV sequence using the ART simulation tool (version GreatSmokyMountains-04-17-2016, NIH).
